# Review of Mobile DNA - finding treasure in junk by Haig H Kazazian

**DOI:** 10.1186/1759-8753-3-16

**Published:** 2012-10-03

**Authors:** Cédric Feschotte

**Affiliations:** 1 Department of Human Genetics, University of Utah, 15 N 2030 E Rm 2100, Salt Lake City, UT, 84112, USA

## Book details

Kazazian, HH

Mobile DNA - finding treasure in junk

FT Press; 2011

288 pages

ISBN: 978-0-13-707062-6

In his celebrated 1970 book, Jacques Monod follows in the footsteps of Democritus who allegedly around 400 BC argued: ‘everything existing in the universe is the fruit of chance and necessity’. ‘Chance and necessity’ also describes how many of us got drawn to the field of mobile genetic elements, and the adage could have served as subtitle to Haig Kazazian’s new book *Mobile DNA - Finding treasure in junk*. Not only does the book provides an historical, detailed, and up-to-date account of the study of the LINE-1 (L1) element, the only transposable element known to be autonomously jumping in humans, it is also a beautiful manifesto for the *ad-lib* path that we all cherish in the basic science enterprise. Kazazian takes us on a personal tour of the twisty route that led him to previously unexplored genomic territories and, along the way, to groundbreaking discoveries.

As the author warns in his introductory chapter, the book does not provide a broad, balanced, or comprehensive overview of the entire field of mobile DNA research. Nor does it attempt to synthesize all the profound implications of this field for understanding genome function and evolution in virtually all branches of life’s tree. While keeping this in mind, Kazazian’s book is a must-read and a trove of information for anyone involved in the study of mobile elements. The book is also highly recommended to anyone even remotely engaged in human genome research. Part of this community continues to ignore the crucial impact of mobile elements in shaping our genome and thereby our biology, as well as that of our ancestors and distant relatives. After over two decades of research on L1 and its allies, the pioneering works of Kazazian and colleagues have illustrated both the destructive and constructive facets of transposition in the genome. This double-edge sword of mobile element activity is recapitulated in vivid colors in this widely accessible book.

The organization of the book is somewhat unorthodox. Following a brief introduction, the book begins with five concise chapters providing background information on the various types and abundance of mobile DNA in diverse organisms. These chapters feel more like a compilation of ‘further reading’ material rather than a real effort to synthesize what unifies or distinguishes the various forms of mobile DNA populating genomes. With Chapter 7, aptly entitled ‘The prologue’, the book takes a sharp turn in style and content. One feels this chapter should and might once have been the opening chapter. It begins with a line that struck me as a classic quote: ‘Rarely does one find Maxine Singer outside a public place without a lit cigarette’. As we get into the true ‘meat’ of the book, the tone becomes decidedly more personal and more entertaining. Through a series of thrilling chapters, Kazazian recounts the unforeseen and convoluted cascade of events that led him to enter, not without some hesitation, the burgeoning field of mobile DNA and the ensuing two decades of seminal discoveries on L1 elements achieved in his laboratory (and a few others). This epic covers more than half of the book, and it is not only *la pièce de resistance,* but also the real treat of the book. In particular, one can feel the excitement building up in the chase for the first active human L1 elements in the mid-to-late 1980s. This section is a real page-turner and I have to admit shamelessly I would have gone back to the buffet for seconds.

Throughout this major slice of the book, Kazazian proceeds largely in chronological order through short chapters that are generally constructed with the same mold. Each introduces the cast of characters that succeeded through his lab, the key questions they set out to tackle, the hurdles they encountered along the way while designing, performing, or interpreting the experiments, and how they eventually succeeded to deliver what has now become classic L1 literature. The style is direct, crisp, and effective, and the juicy anecdotes that punctuate the technical descriptions of key experiments make this part of the book the most informative and enjoyable. The author pays an enormous tribute to the members of his laboratory and he never misses an opportunity to highlight their independence, ingenuity, and exemplary work ethics. He also gives plenty of credit to collaborators and other scientists, notably Maxine Singer and Jef Boeke, but also Tom Eickbush, and later John Moran, who acted as catalysts for Kazazian’s research and produced many crucial tools and concepts to the field.

Perhaps what I would retain as the most important lesson in this saga is Kazazian’s role as an inspired and inspiring leader. His passion for the topic and for science transpires through every page (pore) of this book and it has been contagious. His laboratory has produced not only keystones to our current appreciation of the tremendous influence of L1 in shaping, for better or worse, our genome, but also a crop of creative and dedicated followers that extend far beyond Kazazian’s direct mentees. The book’s Preface, ‘Thoughts on doing science’, lucidly encapsulates what basic science is all about: training, creativity, and perseverance. Without any doubt, scientists at any stage of their career, and not just those interested in mobile DNA, will find something precious to learn from this opus.

## Competing interests

The author declares no competing interests.

